# A Novel Necroptosis-Associated lncRNA Signature Can Impact the Immune Status and Predict the Outcome of Breast Cancer

**DOI:** 10.1155/2022/3143511

**Published:** 2022-05-05

**Authors:** Xin Zhang, Xingda Zhang, Guozheng Li, Yi Hao, Lei Liu, Lei Zhang, Yihai Chen, Jiale Wu, Xinheng Wang, Shuai Yang, Shouping Xu

**Affiliations:** Department of Breast Surgery, Harbin Medical University Cancer Hospital, Harbin 150081, China

## Abstract

Breast cancer (BRCA) is one of the leading causes of death among women worldwide, and drug resistance often leads to a poor prognosis. Necroptosis is a type of programmed cell death (PCD) and exhibits regulatory effects on tumor progression, but few studies have focused on the relationships between necroptosis-associated lncRNAs and BRCA. In this study, we established a signature basis of 7 necroptosis-related lncRNAs associated with prognosis and divided BRCA patients into high- and low-risk groups. Kaplan-Meier curves all showed an adverse prognosis for patients in the high-risk group. Cox assays confirmed that risk score was an independent prognostic factor for BRCA patients. The receiver operating characteristic (ROC) curve proved the predictive accuracy of the signature and the area under the curve (AUC) values of the risk score reached 0.722. The nomogram relatively accurately predicted the prognosis of the patients. GSEA analysis suggested that the related signaling pathways and biological processes enriched in the high- and low-risk groups may influence the tumor microenvironment (TME) of BRCA. ssGSEA showed the difference in immune cell infiltration, immune pathway activation, and immune checkpoint expression between the two risk groups, with the low-risk group more suitable for immunotherapy. According to the significant difference in IC50 between risk groups, patients can be guided for an individualized treatment plan. Overall, the authors established a prognostic signature consisting of 7 necroptosis-associated lncRNAs that can independently predict the clinical outcome of BRCA patients. The difference in the tumor immune microenvironment between the low- and high-risk populations may be the reason for the resistance to immunotherapy in some patients.

## 1. Introduction

The latest global cancer burden data for 2020 shows that the incidence of breast cancer (BRCA) has overtaken lung carcinoma as the most common cancer worldwide, and it is one of the leading causes of cancer death among women [1]. With the development of modern medicine, more sensitive and efficient imaging techniques are emerging, which has led to a distinct increase in the rate of early diagnosis of BRCA and a drastic reduction in the mortality rate, but the prognosis for patients remains poor for a variety of reasons [2, 3]. Traditional treatments for breast cancer, including surgery, targeted therapy, endocrine therapy, radiotherapy, and chemotherapy, have greatly extended patient survival [4]. In addition, there are also some emerging treatment methods, such as immunotherapy, which are gradually introduced in clinical treatment [5]. However, the cure rate remains low due to drug resistance for a variety of reasons [6]. Therefore, finding a reliable prognostic signature and new therapeutic targets are the best ways to achieve a cure for BRCA.

Necroptosis is a new form of programmed cell death (PCD) mediated mainly by mixed lineage kinase domain-like protain [7]. After the tumor necrosis factor binds to its receptor (TNFR1), RIPK1 is activated, and the activated RIPK1 forms a complex with receptor-interacting serine-threonine kinase 3 (RIPK3) [8]. Then, MLKL is phosphorylated and recruited into a complex termed the necrosome through its interaction with RIPK3 [9]. MLKL moves to the plasma membrane to form pores, and then, the membrane is destroyed [10]. Necroptosis eventually leads to swelling of organelles, cell membrane rupture, and release of cytoplasmic contents [11]. More and more evidence indicates that necroptosis is involved in the progression of several types of cancers [12]. After the rupture of the cell membrane, its contents are released, exposing damage-related molecular patterns (DAMP), which induce immunogenic cell death (ICD), promoting the phagocytosis and processing of tumor antigens by dendritic cells (DC) and the activation and proliferation of antitumor T cells [13]. At the same time, it can change the tumor microenvironment (TME), promote the increase of tumor-infiltrating lymphocytes (TILs), and ultimately make the tumor more sensitive to immune checkpoint inhibitors (ICI) [14].

Long noncoding RNA, a kind of transcript longer than 200 bp, does not have the function of an encoding protein [15]. Although lncRNAs cannot be translated into proteins, they still exhibited a regulatory effect on gene expression and several physiological processes such as cell proliferation, differentiation, migration, and apoptosis [16]. According to previous research, lncRNAs can cause breast cancer and its development [17]. For example, in triple-negative breast cancer (TNBC), LINC00096 can inhibit the epithelial-mesenchymal transition (EMT) process by regulating the miR-383-5p/RBM3 axis [18]. In addition, LINC00339 plays a carcinogenic role by regulating the miR-377-3p/HOXC6 axis, which may be a pathogenic factor for breast cancer [19]. Similarly, lncRNA HEIH, as an oncogenic noncoding RNA, is involved in SOCS1-regulated cell proliferation and apoptosis by serving as a sponge for miR-4458 [20]. According to the findings above, it seems clear that lncRNAs play a significant role in BRCA. However, few studies have focused on necroptosis-related lncRNAs, and the mechanism has not been clarified yet. Therefore, the authors need to further characterize these molecules.

In this study, we aimed to develop a novel prognostic model for BC. This work discovered seven lncRNAs linked to necroptosis associated with BRCA and developed a prediction signature that can accurately assess patient prognosis. Then, to better predict patient survivals, we developed a nomogram. In addition, we conducted a differential analysis of TME and drug sensitivity between low-risk and high-risk groups, in order to find a more appropriate treatment for BRCA and offer more valuable insights for clinical treatment selection.

## 2. Materials and Methods

### 2.1. Data Acquisition and Information Extraction

We obtained the transcriptome information of 1208 BRCA patients from The Cancer Genome Atlas (TCGA) website (https://portal.gdc.cancer.gov/) including mRNA and lncRNA levels. Additionally, we gathered clinical data on BRCA patients from the same website, eliminating patients who had missing follow-up information or pathological data that was incomplete, resulting in a total of 848 patients for the following analysis. Necroptosis-associated genes were downloaded from the KEGG website, (https://www.kegg.jp/), and ultimately, 157 genes were obtained by referring to published literature [21, 22]. Finally, 140 genes associated with necroptosis were retrieved from the BRCA mRNA expression data available in TCGA (Table S1).

### 2.2. Building a Predictive Risk Signature Based on lncRNAs Associated with Necroptosis

Pearson correlation analysis was conducted for 140 necroptosis-related genes to examine the relationships between gene and lncRNA data expressed in all cases. Then, a total of 1241 necroptosis-related lncRNAs were identified based on Pearson correlation coefficients greater than 0.3 and *P* values less than 0.05 (*R* > 0.3, *P* < 0.05). Based on univariate assays, 61 lncRNAs related to necroptosis were identified to be distinctly related to prognosis in BRCA patients, which were identified as candidate lncRNAs (Table S2). And then, these lncRNAs were included in multivariate assays to calculate risk scores to construct the risk signature (Table S3). The following formula was used for each patient's risk score.

(1) $$\begin{array}{c} \text{Risk Score}=\text{explncRNA}1*\text{coef lncRNA}1+\text{explncRNA}2*\text{coef lncRNA}2+\cdots +\text{explncRNAi}*\text{coef lncRNAi}. \end{array}$$

exp represents the gene expression and coef represents the coefficient value. Based on the median risk score, BRCA patients in the TCGA datasets were split into high-risk and low-risk categories. The difference in overall survival (OS) was examined utilizing the Kaplan-Meier assays.

### 2.3. Verification of the Necroptosis-Associated lncRNA Risk Signature

We separated the TCGA database cohorts randomly into two groups of about 1 : 1 ratio, called the training set and the testing set, to further evaluate the accuracy of this signature. This algorithm was used to split patients into high- and low-risk groups based on their risk ratings in both training and testing sets. After that, an OS comparison using a Kaplan-Meier analysis was carried out between the two groups. Survival curves were generated, and the area under the curve was calculated using time-dependent receiver operating characteristic (ROC) curves. In addition, univariate and multivariate assays were conducted for the examination of whether risk scores and other possible characteristics were independent variables in predicting prognosis, to determine prognostic indicators.

### 2.4. Bioinformation Analysis

The mRNA-lncRNA coexpression network between seven necroptosis-related lncRNAs and their corresponding necroptosis-related genes was constructed by the use of the Cytoscape program 3.7.2 [23]. The R package “ggalluvial” was used to construct a Sankey diagram to prove the relationship between necroptosis-related lncRNAs and their corresponding genes. Using the R package “scatterplot3d,” a principal component analysis (PCA) diagram was created to examine the distribution of patients under various scenarios.

### 2.5. Construction of Nomogram

We created a nomogram for BRCA patients using the R package “rms,” which combined risk scores with clinical factors such as age, stage, and TNM stage to produce predictions for 1-, 3-, and 5-year survivals. To evaluate the nomogram's accuracy in predicting both expected and actual survival rates, we performed a calibration curve study.

### 2.6. Gene Set and Function Enrichment Analysis (GSEA)

GSEA was used to analyze which cell functions and cell signaling pathways were mainly enriched between high-risk and low-risk patients [24]. GSEA 4.1.0 was used to perform the GSEA. Statistical significance was defined as *P* < 0.05 and a FDR < 0.25. The Gene Ontology (GO) assays were conducted from the perspective of biological functions, and the Kyoto Encyclopedia of Genes and Genomes (KEGG) assays were performed from the perspective of signaling pathways.

### 2.7. Analysis of Tumor Microenvironment and Clinical Treatment Response in the Prognostic Risk Signature

We used the “GSVA” package by single-sample gene set enrichment analysis (ssGSEA) to calculate the immune cells' infiltration scores and the activities of pathways involved in immune function [25]. CIBERSORT algorithm was applied to calculate levels of immune cell infiltration in all cases and examined the relationships between the risk score and the immune checkpoints expression levels [26]. Besides, we compared the IC50 of clinically common drugs for BRCA to observe the sensitivity of different groups of patients to the drug using the R package “pRRophetic.”

### 2.8. Statistical Analysis

All statistical assays were conducted with the use of R software (version 3.6.2). The Wilcoxon test was used to compare proportion of tumor-infiltrating immune cells and expression levels of immune checkpoints between two risk groups. Spearman correlation analysis was used to identify the correlation between tumor-infiltrating immune cells. The forest maps are drawn using the R package “ggplot2.” The R package “survival rock” is applied to plot the ROC curve and determine the area under curve (AUC) value [27]. *P* < 0.05 was considered statistically significant.

## 3. Results

### 3.1. Necroptosis-Associated lncRNAs with Prognostic Value in BRCA Patients Were Identified

**Figure 1:**
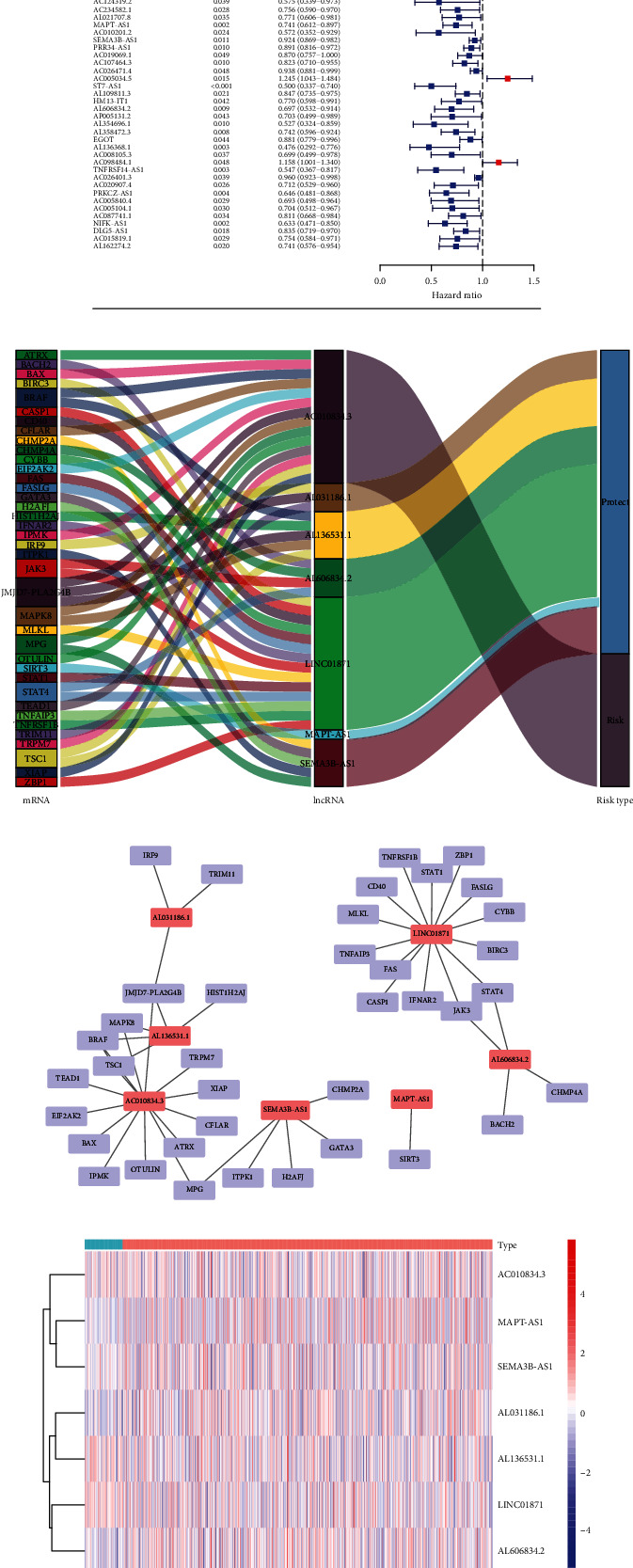
Identification of necroptosis-related lncRNAs with predictive prognostic values in BRCA. (a) The forest plot shows the HR (95% CI) and *P* values (*P* < 0.05) of selected lncRNAs determined using univariate analysis. (b) The Sankey diagram shows the association between lncRNAs, mRNA, and risk types. (c) lncRNA-mRNA coexpression network of necroptosis-associated lncRNAs and corresponding genes. (d) Expression levels of 7 lncRNAs associated with necroptosis in tumor tissue and normal tissue.

We first selected 157 genes related to necroptosis, and according to the expression data obtained in TCGA, a total of 140 genes were expressed. Then, 1241 lncRNAs associated with necroptosis were analyzed by Pearson correlation analysis (*R* > 0.3, *P* < 0.05). After that, 61 lncRNAs were found to be prognostic by univariate assays, suggesting that these lncRNAs may have predictive values for outcomes of BRCA patients. Among them, the HR of 6 lncRNAs was greater than 1.0, while the HR of 55 lncRNAs was less than 1.0 (Figure 1(a)).

### 3.2. Identification of Necroptosis-Associated lncRNAs Associated with Prognosis in Breast Cancer

We used multivariate assays for the identification of 7 lncRNAs associated with necroptosis ultimately (AC010834.3, AL031186.1, AL136531.1, LINC01871, MAPT-AS1, SEMA3B-AS1, and AL606834.2) and applied them to develop a predictive prognostic risk signature of breast cancer. Among them, 1 lncRNA was an unfavorable prognostic factor, while the remaining 6 lncRNAs were favorable prognostic factors (Figure 1(b)). The expression relationship between these 7 lncRNAs and necroptosis-related genes is shown in Figure 1(c). In addition, according to the transcriptome information extracted from the TCGA database, we can also see the expressions of the 7 lncRNAs in normal tissues and tumor specimens (Figure 1(d)). Then, based on the risk score formula, we calculated the risk scores of each patient, ranked them, and used the median as the threshold, thus dividing the patients into two groups (high-risk group: *n* = 511, and low-risk group: *n* = 511). The median value of the risk score is 1.18178.

### 3.3. Correlation between Prediction Signature and Outcome of Patients

**Figure 2:**
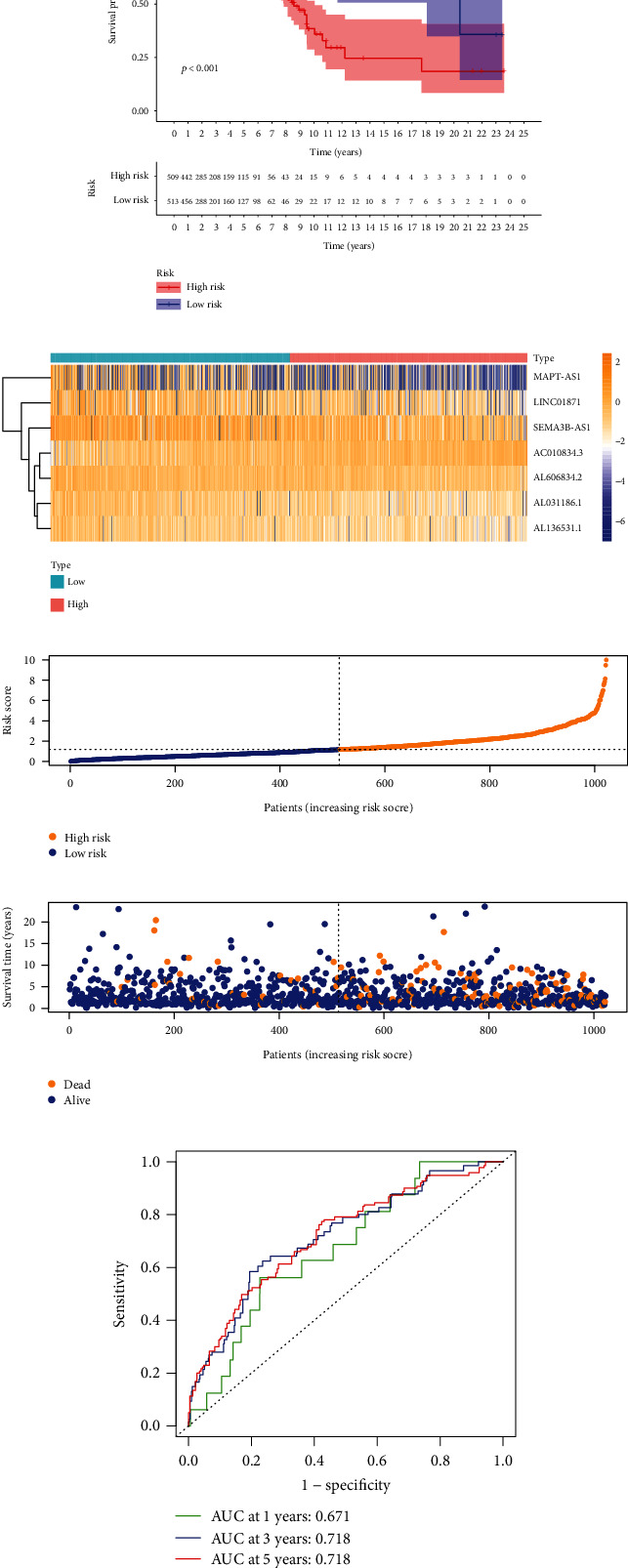
Correlation between predictive risk signature constructed by 7 necroptosis-associated lncRNAs and prognosis of BRCA patients. (a) Kaplan-Meier curve analyzed OS rates in high- and low-risk BRCA patients (b) Heat map of expression of 7 lncRNAs. (c) Risk score distribution in BRCA patients (d) Survival status of patients with different risk scores. (e) ROC curve and area under the curve at long-term survivals.

Kaplan-Meier analysis was performed to compare the OS times of patients in the low- and high-risk groups based on individual risk scores. Compared with the high-risk group, the OS time in the low-risk group was distinctly longer (Figure 2(a)). The expression levels of the 7 lncRNAs in the high-risk and low-risk groups are shown in Figure 2(b). The risk scores of the two groups are displayed in Figure 2(c). More patients died as their risk scores rose (Figure 2(d)). Moreover, according to the ROC curve analysis, the AUC values were: 1-year survival of 0.671, 3-year survival of 0.718, and 5-year survival of 0.718, indicating that the signature exhibited a strong ability in predicting outcomes of BRCA patients (Figure 2(e)). The accuracy of the signature was tested using training and testing sets (Supplement Figure S1A-S1J). Table S4 displays the individual patient data from the two groups.

### 3.4. Cell Function and Pathway Enrichment in GSEA

**Figure 3:**
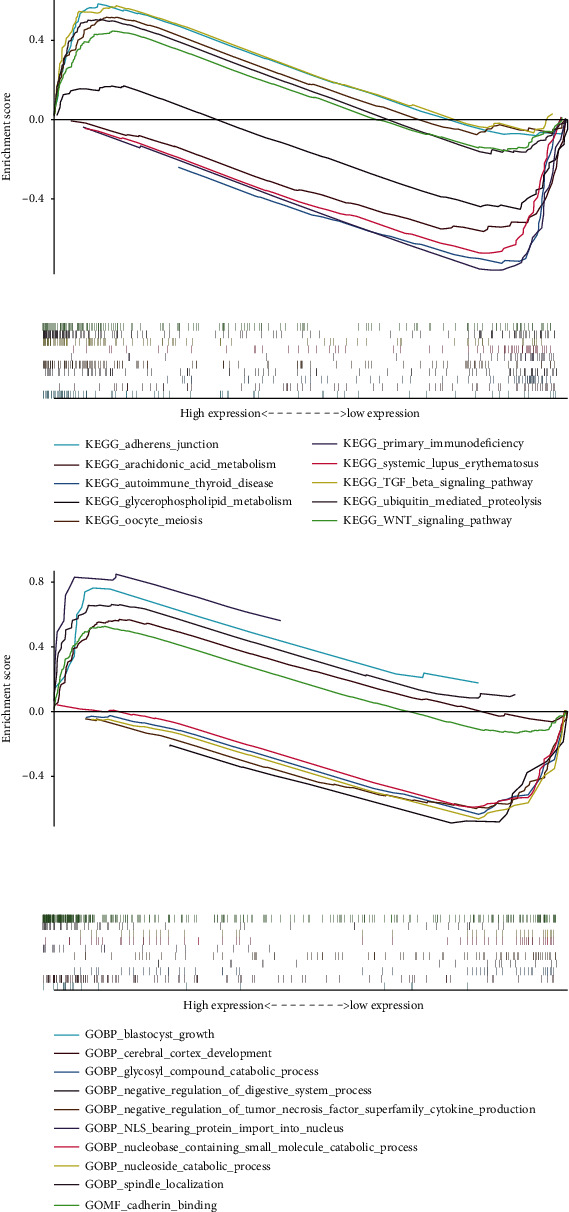
GSEA of the dysregulated genes between low-risk and high-risk groups. (a) GSEA results of differential enrichment of KEGG genes and lncRNA expression associated with necroptosis. (b) GSEA data of differential enrichment of GO and lncRNA expressions associated with necroptosis.

GSEA was used for further analysis and functional annotation. KEGG pathways are being identified to study necroptosis-related signaling pathways. The data of GSEA revealed that TGF-*β* signaling pathway, adherens junction, ubiquitin-mediated proteolysis, Wnt signaling pathway, and oocyte meiosis were enriched in the high-risk group (Figure 3(a)), while some other pathways were enriched in the low-risk group, including arachidonic acid metabolism, autoimmune thyroid disease, systemic lupus erythematosus, glycerophospholipid metabolism, and primary immunodeficiency. In addition, we further investigated the biological processes of lncRNAs associated with necroptosis. GO enrichment results indicate that cerebral cortex development, NLS bearing protein import into nucleus, cadherin binding, spindle localization, and blastocyst growth were enriched in groups with higher risk. However, negative regulation of the digestive system process, glycosyl compound catabolic process, negative regulation of tumor necrosis factor superfamily cytokine production, nucleoside catabolic process, and nucleobase-containing small molecule catabolic process were enriched in the low-risk group (Figure 3(b)).

### 3.5. Comparative Study of High- and Low-Risk Groups

**Figure 4:**
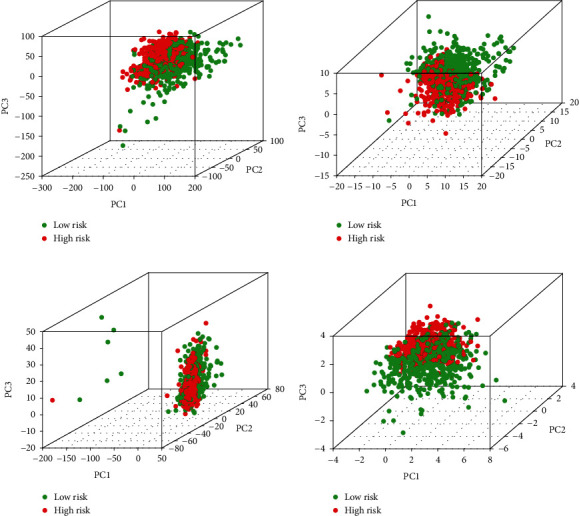
Principal component analysis. A comparison of gene expression levels in low- and high-risk populations, as determined by the expression of all examined genes (a), necroptosis-related genes (b), and lncRNAs (c) and the 7 lncRNAs of the prognostic signature (d).

Figures 4(a)–4(d) exhibited the results of principal component analysis. The four graphs represent the expression levels of different gene types in high-risk and low-risk groups. It was clear that in both groups of patients, the expression of all examined genes, necroptosis-associated genes, and lncRNAs could not effectively discriminate between the low- and high-risk groups (Figures 4(a)–4(c)). However, there was a distinct difference between the two groups in the expressions of the 7 lncRNAs used in the risk signature (Figure 4(d)). Through this, we can determine that the necroptosis signature well distinguishes populations at different risk.

### 3.6. Assessment of Risk Score as Independent Prognostic Factor and Prediction of the Clinical Survival

**Figure 5:**
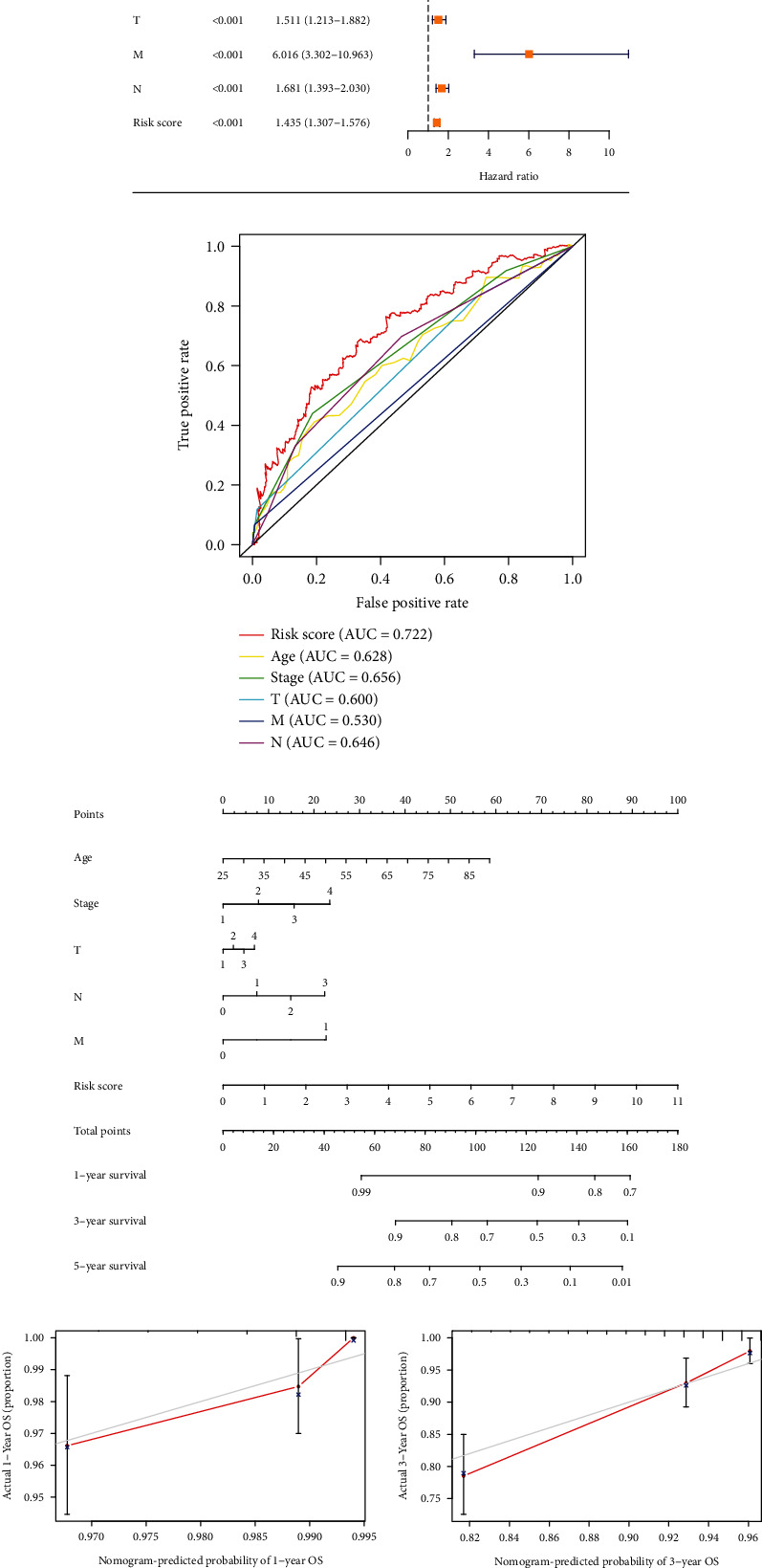
Risk scores became independent predictors of prognosis and constructed patient prognostic nomogram. Univariate (a) and multivariate (b) analyses were performed to observe the association between clinical-pathological elements and overall survival. (c) The ROC curve of age; stage; T, N, M stage; and risk score with OS for BRCA cohorts. A clinical prognostic nomogram (d) was developed to predict survival time. Calibration curves showing the nomogram projections for 1-year (e), 3-year (f), and 5-year (g) survivals.

Risk scores for BRCA patients were examined using univariate and multivariate assays to establish whether or not the score was an independent predictor of OS in these individuals. In the univariate analysis, the HR (95% CI) of the risk score was 1.435 (1.307-1.576) (*P* < 0.001, Figure 5(a)); also, in multivariate assays, the HR (95% CI) for the risk score was 1.357 (1.238-1.486) (*P* < 0.001, Figure 5(b)). Besides, AUC values of the ROC curve were used to determine the accuracy of the signature (Figure 5(c)). Among them, the AUC value of the risk score (0.722) was significantly higher than other clinical parameters, further demonstrating the excellent predictive ability of the signature for patients. To further predict the outcome of patients with BRCA, we constructed a nomogram that predicted the probability of survivals of cases at 1, 3, and 5 years (Figure 5(d)). Calibration curves showed good agreement between actual OS rates and predicted 1-, 3-, and 5-year survival rates (Figures 5(e)–5(g)).

### 3.7. Prognosis Was Predicted by Combining Different Clinical-Pathological Variables

**Figure 6:**
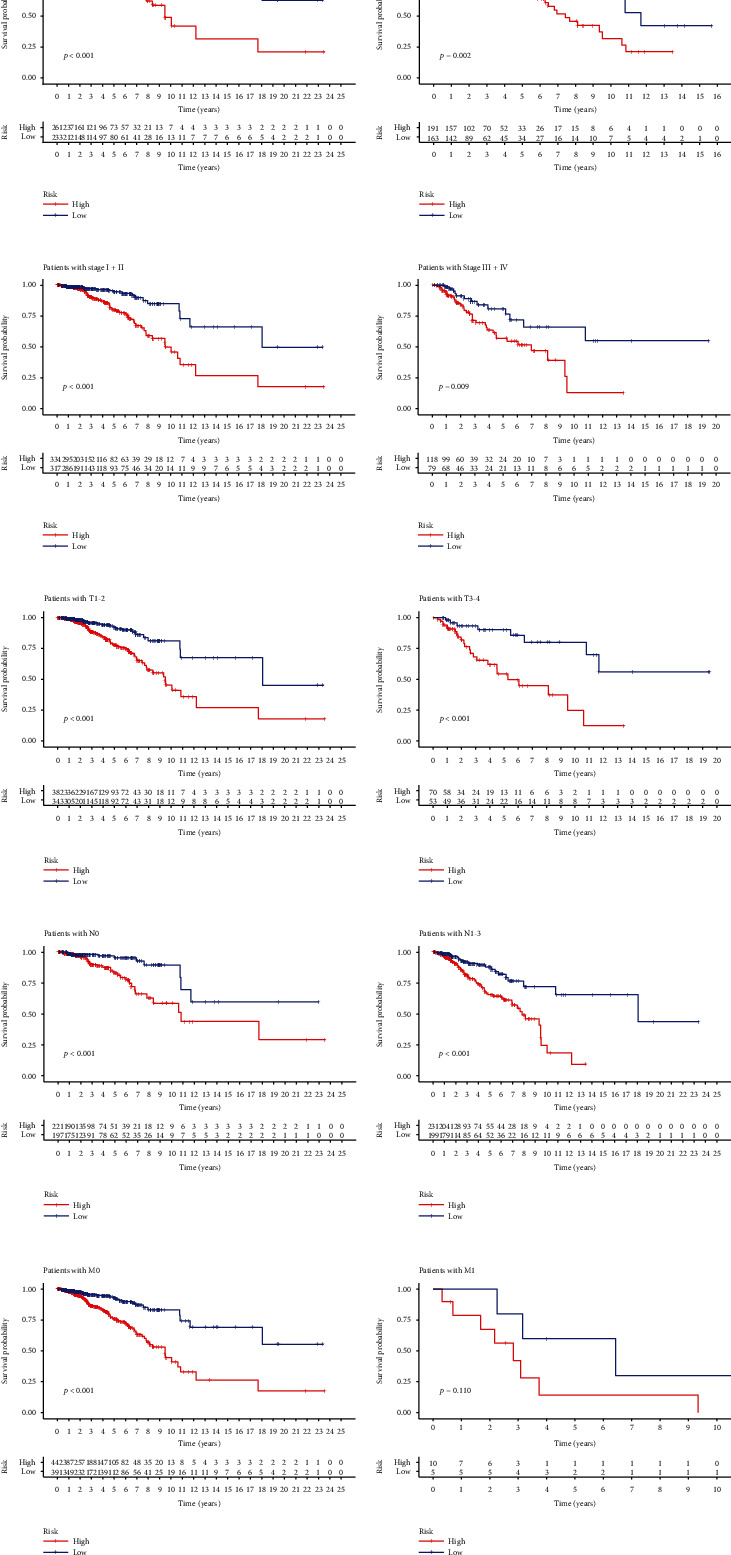
Kaplan-Meier assays in patients (high and low groups) sequenced according to different clinical-pathological ingredients: (a, b) age; (c, d) stage; (e, f) T stage; (g, h) N stage; (i, j) M stage.

**Figure 7:**
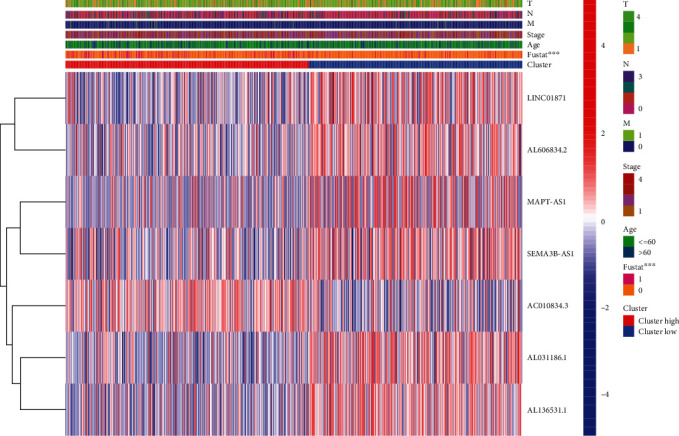
A heat map of the distribution of seven prognostic necroptosis-related lncRNAs and clinical factors was created.

To investigate the relationships between predictive characteristics and outcomes of BRCA patients classified based on several clinical-pathological features, they were grouped by age, survival status, stage, and TNM stage (Table S5). For each different classification, the OS of patients with high-risk scores was distinctly shorter (Figures 6(a)–6(j)). Our findings suggested that risk scores can predict the survival time of BRCA patients regardless of other clinical elements. In addition, for each of the seven prognostic lncRNAs and clinical components, we examined the distribution of expression in the high-risk and low-risk groups (Figure 7).

### 3.8. Correlation Analysis of Risk Score and 7 Prognostic lncRNAs with Clinical-Pathological Factors

**Figure 8:**
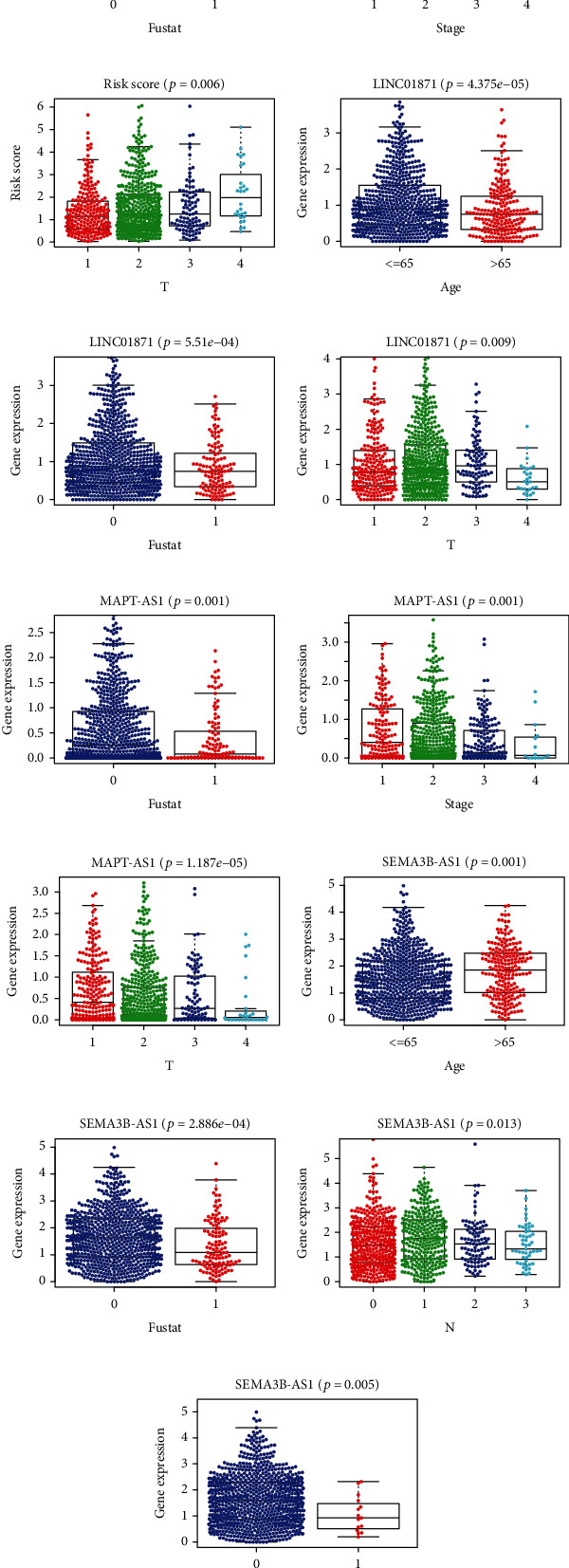
Risk scores and 7 necroptosis-associated lncRNAs were associated with clinical-pathological features in BRCA patients. Correlation between risk score (a–c), LINC01871 (d–f), MAPT-AS1 (g–i), and SEMA3B-AS1 (j–m) and clinical-pathological features.

To examine the roles of the risk score in breast cancer development, our group carried out correlation assays between the risk score and clinical-pathological elements. We also evaluated the role of the 7 lncRNAs in disease development. As shown in Figure 8(a), risk scores were distinctly related to patient survival status. At the same time, the risk score is also correlated with stage and T stage (Figures 8(b) and 8(c)). We observed that risk scores are closely correlated with patient outcomes. In addition, the different lncRNA was also correlated with clinical-pathological ingredients. Among the 7 necroptosis-related lncRNAs, 3 protective lncRNAs played a significant role. LINC01871 was associated with patients' age, survival status, and T stage (Figures 8(d)–8(f)). MAPT-AS1 was associated with patients' survival status, stage, and T stage (Figures 8(g)–8(i) SEMA3B-AS1 was associated with patients' age, survival status, and N and M stages (Figures 8(j)–8(m)). In addition, it can be seen from the figure that the expressions of these 3 lncRNAs are reduced in patients with older age, higher stage, and death, which is consistent with our previous findings. This may be a new therapeutic target, and we need further experimental validation.

### 3.9. Analysis of Tumor Microenvironment of Breast Cancer

**Figure 9:**
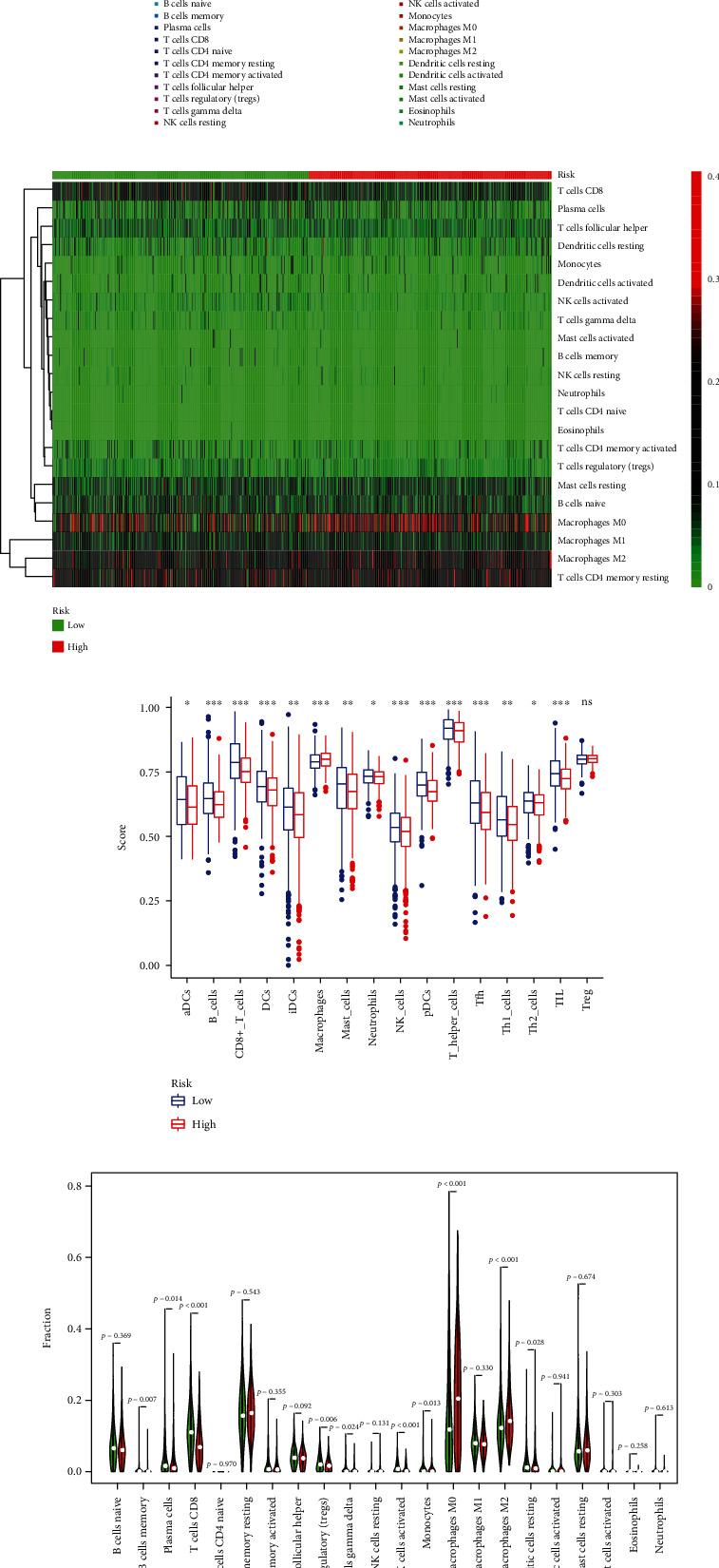
Immune-infiltrating cell score analysis of tumor microenvironment in patients with different risks. (a) The proportion of immune-infiltrating cells in tumors. (b) Heat map of tumor immune-infiltrating cell expression. (c, d) Showing levels of infiltration of immune cells.

**Figure 10:**
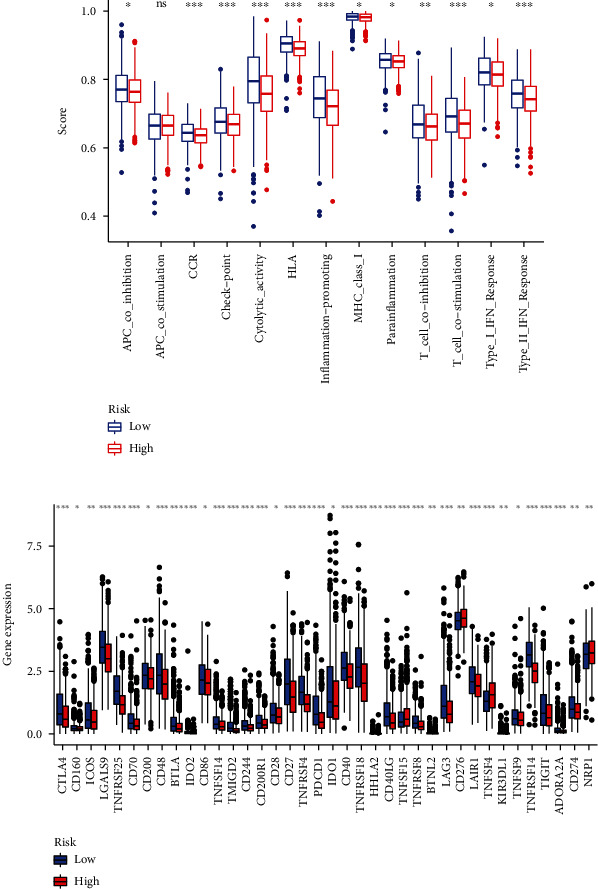
Analysis of immune-related functions and immune checkpoint expression in patients at high and low risk. (a) Correlations between predictive signature and immune-related functions. (b) Differences in immune checkpoint expression levels among different groups. ^∗∗∗^*P* < 0.001, ^∗∗^*P* < 0.01, ^∗^*P* < 0.05.

BRCA patients with necroptosis-related lncRNAs and TME were analyzed by the use of the CIBERSORT algorithm, which calculated the fraction of different immune cells. The results showed that the proportion of different tumor-infiltrating immune cells was significantly different between the two groups (Figures 9(a) and 9(b)). As shown in Figures 9(c) and 9(d), the CD8+ T cell, activated dendritic cells (aDCs), DC cell, NK cell, and tumor-infiltrating lymphocyte (TIL) were higher in the low-risk group, while the M0 macrophage cell, M2 macrophage cell, and DC resting cell were higher in the high-risk group. In addition, we analyzed the differences in immune function and immune checkpoint between the two groups. As shown in Figure 10(a), the checkpoint, chemokine receptor, cytolytic activity, human leukocyte antigen, T cell costimulation, inflammation promotion, and type II IFN response were significant differences, which shows that tumors in high-risk groups grow in immunosuppressive microenvironments. Moreover, as shown in Figure 10(b), the expressions of immune checkpoints, including CD274, CTLA4, LAG3, and TIGIT, are generally low in the high-risk group, which partly explains the lack of effects of ICI in BRCA, especially in TNBC.

### 3.10. Relationship between Prognostic Risk Signature and Drugs for Treating BRCA

**Figure 11:**
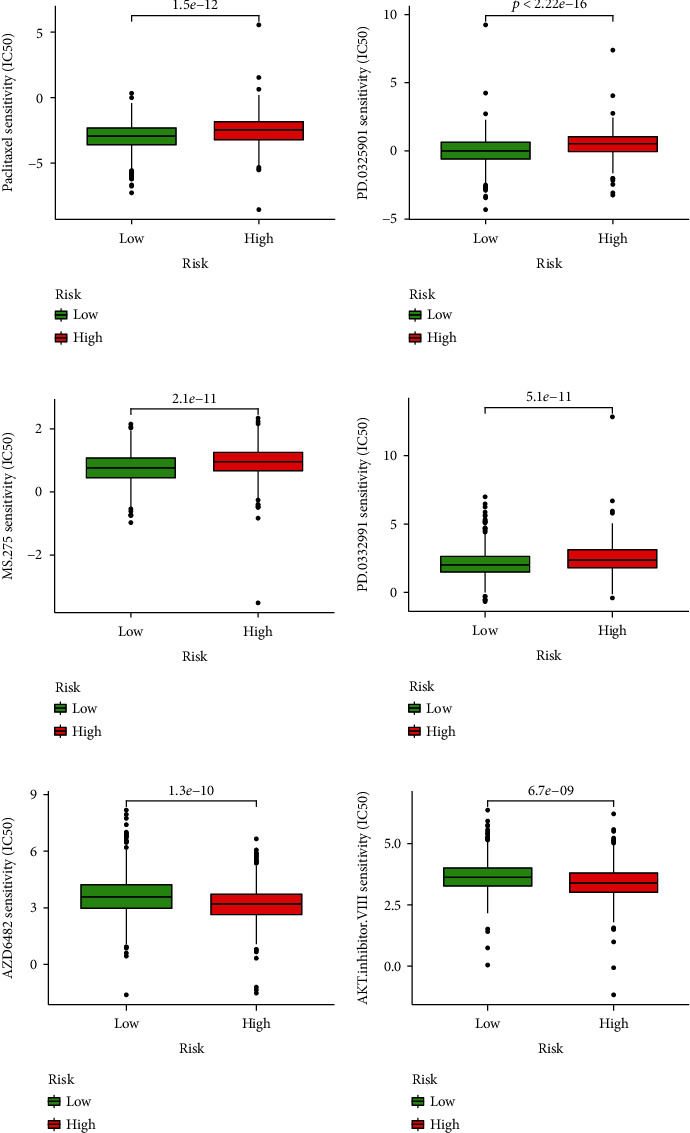
Comparison of drug sensitivity between high-risk and low-risk groups. IC50 of paclitaxel (a), IC50 of CDK4/6 inhibitors (b), HDAC inhibitors (c), MEK inhibitors (d), PI3K inhibitors (e), and Akt inhibitors (f) in high-risk and low-risk populations.

Our group analyzed the relationship between predictive signature and medication in breast cancer patients and determined that paclitaxel, CDK4/6 inhibitors, and HDAC inhibitors recommended in clinical guidelines are more suitable for high-risk patients based on the IC50 values of different risk groups (Figures 11(a)–11(c)). Besides, we also studied the relationship between small-molecule inhibitors and BRCA. The IC50 of MEK inhibitors was higher in the high-risk group, while those of PI3K inhibitors and Akt inhibitors were higher in the low-risk group (Figures 11(d)–11(f)). The above findings may help in the search for individualized treatment options.

## 4. Discussion

In this study, 61 lncRNAs associated with necroptosis were identified, of which 6 was a risk factor and the rest were protective factors. Then, multivariate analysis was applied to further identify 7 lncRNAs, i.e., AC010834.3, AL031186.1, AL136531.1, LINC01871, MAPT-AS1, SEMA3B-AS1, and AL606834.2, to have significant correlation with the prognosis of patients. Among them, MAPT-AS1 and SEMA3B-AS1 have been reported already. For example, researchers have found that MAPT-AS1 is overexpressed in breast cancer and that the high expression of MAPT-AS1 is beneficial to patient survival and is probably a potential survival predictive biomarker in breast cancer [28]. In addition, it has been reported that SEMA3B-AS1 is downregulated in hepatocellular carcinoma cells (HCC) and that its overexpression may inhibit the proliferation of HCC cells by upregulating PTEN via the downregulation of miR-718 [29]. Moreover, SEMA3B-AS1 plays a tumor-suppressive role in gastric cardia adenocarcinoma tumorigenesis, and its expression level is coregulated by promoter aberrant hypermethylation and histone modification [30]. The authors demonstrated that the risk signature was more accurate and reliable in predicting BRCA patients' prognosis without considering other clinical-pathological ingredients.

In addition, GO and KEGG analyses using these differentially expressed necroptosis-associated lncRNAs revealed that these lncRNAs were primarily involved in the TGF-*β* signaling pathway, WNT signaling pathway, and negative regulation of TNF superfamily cytokine production. Previous studies have found that TGF-*β* promotes the metastasis of breast cancer cells by inducing epithelial mesenchymal transformation (EMT) [31], thus making these cells lose their cell polarity and adhesion characteristics, and developing the ability of migration and invasion [32]. TGF-*β* can upregulate the expression of CXC chemokine receptor 4 (CXCR4), thereby enhancing the metastatic potential of BRCA [33]. In TNBC, the PD-L1 expression was upregulated through the WNT signaling pathway, and then, CD8+ T cell activation was blocked [34]. Moreover, the WNT signaling pathway stimulates IL-1 *β* production by tumor-associated macrophages (TAM), resulting in phenotypically altered neutrophils producing inducible nitric oxide synthase (iNOS), which inhibits the activity of antitumor CD8+ T cells and drives the metastasis of BRCA [35]. The TNF superfamily of ligands (TNFSF) and receptors (TNFRSF) is cosignaling in regulatory T (Treg) cells to suppress immune responses [36]. In addition, the TNF-related apoptosis-inducing ligand (TRAIL), also a member of the TNF superfamily, can transform the TME into a more immunosuppressive type that promotes tumor growth [37].

In previous studies, cell death is divided into PCD and accidental cell death (ACD) according to genetic control [38], and apoptosis was considered to be the only form of PCD [39]. However, in recent years, many other forms have been found, such as pyroptosis, ferroptosis, and necroptosis [40]. PCD produces inflammatory mediators that activate innate immune responses and change the state of the tumor microenvironment [41]. Induction of immunogenic cell death (ICD) is the premise and basis of tumor immunotherapy [42]. The molecular pathways of necroptosis and apoptosis are very similar, but the morphological changes are quite different [43]. The result of apoptosis is immune tolerance, while necroptosis can activate the immune system to achieve tumor clearance by inducing a robust inflammatory response [44].

The newly emerged ICI is a revolutionary approach to cancer treatment, whose main target is to restore antitumor immunity, yet only works in less than one-third of patients [45]. In addition, secondary drug resistance often occurs in patients who respond to treatment, which further limits the progress of immunotherapy [46]. Tumor cells often develop a variety of mechanisms to achieve immune evasion. First, there is reduced expression of immune checkpoint receptors, such as PD-L1, PD-1, CTLA-4,and TIM-3 [47]. The second is the increase in immunosuppressive cell infiltration in the TME, for example, M2 tumor-associated macrophages, MDSC, and Treg cell [48]. It has been shown that the key to ICI's therapeutic effect is the number of tumor-infiltrating lymphocytes (TIL) [49], and because there is usually less TIL infiltration in breast cancer, it is called a “cold” tumor [50]. Since necroptosis and antitumor immunity are closely linked, necroptosis transforming “cold” tumors into “hot” tumor in BRCA is the hinge to make immunotherapy more sensitive.

According to subsequent results of tumor microenvironment analysis, CD8+ T cell, NK cell, TIL, and other tumor-killing immune cells scored higher in the low-risk group, while M2-type macrophages and other immunosuppressive cells scored higher in the high-risk group. In addition, the expression of immune cell-related activation pathways was also different between the two groups, with higher expression in the low-risk group. For the expression level of the immune checkpoint, the high-risk group generally expressed less, such as CD274, CTLA4, and LAG3. Studies have shown that the lack of TIL and the deficiency of immune checkpoint expression are one of the reasons that lead to tumor insensitivity to ICI [51]. Inflammatory forms that trigger localized tumor necroptosis alter the tumor microenvironment and enhance the response to ICI [52]. This is consistent with our results.

However, our research has some imperfections. Most of the data analyzed in this study was based on TCGA datasets, and we need more samples for repeated validation. Secondly, we need to conduct some in vivo and in vitro experiments to verify our findings. In addition, the specific mechanism and relationship between these lncRNAs and breast cancer still need further study.

## 5. Conclusion

We identified necroptosis-associated lncRNAs related to the prognosis of breast cancer and established a prognostic risk signature. At the same time, this study found differences in the tumor microenvironment in high- and low-risk groups, providing a reasonable explanation for immune drug resistance. In addition, we performed a drug sensitivity analysis to indicate the direction of individualized treatment for breast cancer patients.

## Data Availability

The authors declare that all the other data supporting the findings of this study are available within the article and its additional files and from the corresponding author upon reasonable request.

## Supplementary Materials

Supplementary 1 Table S1: a list of necroptosis-related genes.

Supplementary 2 Table S2: univariate cox proportional hazard regression analysis results of necroptosis-related lncRNAs in BRCA.

Supplementary 3 Table S3: multivariate Cox proportional hazard regression analysis results of necroptosis-related lncRNAs in BRCA.

Supplementary 4 Table S4: clinical information variables for BRCA patients in the high- and low-risk groups.

Supplementary 5 Table S5: Demographic and clinical characteristics of BRCA patients in TCGA, training set, and testing set.

Supplementary 6 Supplement Figure 1: the training set and testing set are used to examine the veracity of the signature. (A) Kaplan-Meier analyzed OS rates in the high - and low-risk training set patients. (B) Heat map of expression of 7 lncRNAs in the training set. (C) Risk score distribution in training set patients. (D) Survival status of training set patients with different risk scores. (E) ROC curve and AUC value at 1-year, 3-year, and 5-year survival for the training set. (F) Kaplan-Meier analyzed OS rates in high- and low-risk testing set patients. (G) Heat map of expression of 7 lncRNAs in the testing set. (H) Risk score distribution in the testing set patients. (I) Survival status of the testing set patients with different risk scores. (J) ROC curve and AUC value at 1-year, 3-year, and 5-year survival for the testing set.
