# Supplementary material for: A Novel Necroptosis-Associated lncRNA Signature Can Impact the Immune Status and Predict the Outcome of Breast Cancer
Source: J Immunol Res. 2022 May 5;2022:3143511. doi: 10.1155/2022/3143511 (PMC9107037; doi:10.1155/2022/3143511)
Supplement: Supplementary 1 — Table S1: a list of necroptosis-related genes. [file 3143511.f1.docx]

**Table S1** A list of necroptosis-related genes.

| A list of necroptosis-related genes |
| --- |
| AIFM1 |
| ALDH2 |
| ALK |
| ALOX15 |
| APP |
| ATRX |
| AXL |
| BACH2 |
| BAX |
| BCL2 |
| BCL2L11 |
| BID |
| BIRC2 |
| BIRC3 |
| BNIP3 |
| BRAF |
| CAMK2 |
| CAPN1 |
| CAPN2 |
| CASP1 |
| CASP8 |
| CD40 |
| CDKN2A |
| CFLAR |
| CHMP1 |
| CHMP2A |
| CHMP2B |
| CHMP4A |
| CHMP4B |
| CHMP5 |
| CHMP6 |
| CHMP7 |
| CXCL1 |
| CYLD |
| DDX58 |
| DIABLO |
| DNM1L |
| DNMT1 |
| EGFR |
| EIF2AK2 |
| EZH2 |
| FADD |
| FAF1 |
| FAS |
| FASLG |
| FLT3 |
| FTH1 |
| FTL |
| GATA3 |
| GLUD1 |
| GLUD2 |
| GLUL |
| H2A |
| HAT1 |
| HDAC9 |
| HMGB1 |
| HSP90A |
| HSP90AA1 |
| HSPA4 |
| ID1 |
| IDH1 |
| IDH2 |
| IFNA |
| IFNAR1 |
| IFNAR2 |
| IFNB |
| IFNG |
| IFNGR1 |
| IFNGR2 |
| IL1A |
| IL1B |
| IL33 |
| IPMK |
| IRF9 |
| ITPK1 |
| JAK1 |
| JAK2 |
| JAK3 |
| JNK |
| KLF9 |
| LEF1 |
| MAP3K7 |
| MAPK8 |
| MLKL |
| MPG |
| MYC |
| MYCN |
| NDRG2 |
| NLRP3 |
| NOX2 |
| NR2C2 |
| OTULIN |
| PANX1 |
| PARP2 |
| PARP3 |
| PARP4 |
| PGAM5 |
| PLA2G4 |
| PLK1 |
| PPIA |
| PPID |
| PYCARD |
| PYG |
| RBCK1 |
| RIPK1 |
| RIPK3 |
| RNF31 |
| SHARPIN |
| SIRT1 |
| SIRT2 |
| SIRT3 |
| SLC25A4S |
| SLC39A7 |
| SMPD1 |
| SPATA2 |
| SQSTM1 |
| STAT1 |
| STAT2 |
| STAT3 |
| STAT4 |
| STAT5A |
| STAT5B |
| STAT6 |
| STUB1 |
| TARDBP |
| TERT |
| TIRP |
| TLR2 |
| TLR3 |
| TLR4 |
| TNF |
| TNFAIP3 |
| TNFRSF10A |
| TNFRSF10B |
| TNFRSF1A |
| TNFRSF1B |
| TNFRSF21 |
| TNFRSF6 |
| TNFSF10 |
| TNFSF6 |
| TRADD |
| TRAF2 |
| TRAF5 |
| TRIF |
| TRIM11 |
| TRPM7 |
| TSC1 |
| TYK2 |
| USP21 |
| USP22 |
| VDAC1 |
| VDAC2 |
| VDAC3 |
| VPS24 |
| VPS4 |
| XIAP |
| ZBP1 |
