# Supplementary material for: A Novel Necroptosis-Associated lncRNA Signature Can Impact the Immune Status and Predict the Outcome of Breast Cancer
Source: J Immunol Res. 2022 May 5;2022:3143511. doi: 10.1155/2022/3143511 (PMC9107037; doi:10.1155/2022/3143511)
Supplement: Supplementary 2 — Table S2: univariate cox proportional hazard regression analysis results of necroptosis-related lncRNAs in BRCA. [file 3143511.f2.docx]

Table S2 Univariate cox proportional hazard regression analysis results of necroptosis-related lncRNAs in BRCA .

| gene | KM | B | SE | HR | HR.95L | HR.95H | pvalue |
| --- | --- | --- | --- | --- | --- | --- | --- |
| AC024361.1 | 0.005056106 | -0.442933281 | 0.212086985 | 0.642150049 | 0.423748088 | 0.973117514 | 0.036757169 |
| AC010834.3 | 0.033135232 | 0.148263902 | 0.073047852 | 1.159818934 | 1.005105734 | 1.338346719 | 0.042388884 |
| LINC02062 | 0.040529113 | -0.573990071 | 0.226207539 | 0.563273448 | 0.361552254 | 0.877541139 | 0.011166367 |
| FLJ42351 | 0.019912326 | -0.518625153 | 0.223574965 | 0.595338485 | 0.384110875 | 0.92272293 | 0.020357522 |
| AL513477.2 | 0.040518245 | -0.459284638 | 0.16473422 | 0.631735403 | 0.45741794 | 0.872483531 | 0.005302895 |
| AC004067.1 | 0.028919634 | -0.40626862 | 0.191737501 | 0.666131207 | 0.457459366 | 0.969989508 | 0.034100669 |
| STAG3L5P-PVRIG2P-PILRB | 0.005389575 | -0.389173595 | 0.164134757 | 0.677616629 | 0.491215765 | 0.934750732 | 0.017737082 |
| SH3BP5-AS1 | 0.00283077 | -0.263501307 | 0.10419308 | 0.768356618 | 0.62643141 | 0.942436607 | 0.011439739 |
| AL031186.1 | 0.022215505 | -0.356541003 | 0.172570754 | 0.700093765 | 0.499187519 | 0.981858043 | 0.038823104 |
| AC061992.1 | 0.025249994 | -0.3681912 | 0.14443767 | 0.691984862 | 0.521375988 | 0.918421753 | 0.010799032 |
| AL136295.7 | 0.003544407 | -0.201944811 | 0.095225586 | 0.817140023 | 0.678016585 | 0.98481045 | 0.033947134 |
| AC103691.1 | 0.021010461 | -0.197037015 | 0.099663859 | 0.821160238 | 0.675451049 | 0.998302003 | 0.048039856 |
| AL022328.3 | 0.012039815 | -0.513854233 | 0.223218287 | 0.598185583 | 0.386217716 | 0.926487774 | 0.021333723 |
| Z68871.1 | 0.007030445 | 0.23247685 | 0.108139311 | 1.261721237 | 1.020739837 | 1.559594739 | 0.0315718 |
| AC005785.1 | 0.045262371 | -0.78620579 | 0.278650203 | 0.455570049 | 0.263856429 | 0.786579542 | 0.004780272 |
| AL136531.1 | 0.006195137 | -0.450421344 | 0.20791522 | 0.637359548 | 0.424039916 | 0.957992815 | 0.030282907 |
| AL122010.1 | 0.004150457 | -0.338665816 | 0.098309856 | 0.712720589 | 0.587811129 | 0.864173224 | 0.00057131 |
| USP30-AS1 | 0.006018108 | -0.238514241 | 0.090265372 | 0.787797469 | 0.660055656 | 0.940261395 | 0.008232879 |
| AL451085.2 | 0.019778723 | -0.459680203 | 0.167447833 | 0.631485561 | 0.45481164 | 0.876789375 | 0.006047138 |
| PCED1B-AS1 | 0.008512477 | -0.099686403 | 0.049176262 | 0.905121217 | 0.82195461 | 0.996702747 | 0.042649686 |
| AC136475.2 | 0.00390729 | -0.232456169 | 0.085871893 | 0.792584488 | 0.669809465 | 0.937863981 | 0.006789199 |
| AC132872.3 | 0.038982921 | -0.261896048 | 0.119612683 | 0.76959102 | 0.608759135 | 0.972914088 | 0.028558043 |
| LINC01786 | 0.018109894 | -0.328733133 | 0.141584428 | 0.719835091 | 0.545401252 | 0.950057517 | 0.020242791 |
| LINC01871 | 0.011846252 | -0.222263368 | 0.066645876 | 0.800704456 | 0.702656669 | 0.912433703 | 0.000853023 |
| AC254562.3 | 0.004426406 | -0.505755488 | 0.199235509 | 0.603049806 | 0.408097195 | 0.891133469 | 0.011133495 |
| OTUD6B-AS1 | 0.022544249 | 0.084608796 | 0.026076881 | 1.088291239 | 1.03406646 | 1.145359478 | 0.001176197 |
| BAIAP2-DT | 0.001637824 | -0.059921338 | 0.026233122 | 0.941838618 | 0.894636927 | 0.991530704 | 0.022360583 |
| AC124319.2 | 0.010820026 | -0.553914785 | 0.268793805 | 0.574695591 | 0.339343992 | 0.973274998 | 0.039327624 |
| AC234582.1 | 0.003877763 | -0.27932369 | 0.127073927 | 0.756295058 | 0.589556918 | 0.970189981 | 0.027940584 |
| AL021707.8 | 0.005352416 | -0.259781685 | 0.122963138 | 0.771219936 | 0.606054716 | 0.981396852 | 0.03462822 |
| MAPT-AS1 | 0.002805298 | -0.299959237 | 0.097371966 | 0.740848419 | 0.612133561 | 0.896628473 | 0.002066185 |
| AC010201.2 | 0.001350943 | -0.5591908 | 0.247835554 | 0.571671473 | 0.351713076 | 0.92919 | 0.024051991 |
| SEMA3B-AS1 | 0.017401495 | -0.078818765 | 0.031156524 | 0.924207408 | 0.869458713 | 0.982403557 | 0.011413809 |
| PRR34-AS1 | 0.036383638 | -0.115617455 | 0.04459714 | 0.890815935 | 0.81625682 | 0.972185482 | 0.009528509 |
| AC019069.1 | 0.035661675 | -0.139332977 | 0.070879737 | 0.869938311 | 0.757103956 | 0.99958884 | 0.049325648 |
| AC107464.3 | 0.00566245 | -0.194223757 | 0.075589939 | 0.823473626 | 0.710080231 | 0.954974922 | 0.010186325 |
| AC026471.4 | 0.026958368 | -0.063781895 | 0.032269629 | 0.938209606 | 0.88070795 | 0.999465559 | 0.048094766 |
| AC005034.5 | 0.048037893 | 0.218768218 | 0.08993201 | 1.244542781 | 1.043420978 | 1.484431276 | 0.014991031 |
| ST7-AS1 | 0.005285496 | -0.694111129 | 0.200615973 | 0.499518258 | 0.337121725 | 0.740143608 | 0.000540377 |
| AL109811.3 | 0.02480344 | -0.166357511 | 0.071976778 | 0.84674346 | 0.735334778 | 0.975031385 | 0.020818142 |
| HM13-IT1 | 0.03756934 | -0.261642363 | 0.128661179 | 0.769786279 | 0.598209866 | 0.990573624 | 0.041994303 |
| AL606834.2 | 0.002486087 | -0.360262203 | 0.138013407 | 0.697493417 | 0.532185317 | 0.914149736 | 0.009045172 |
| AP005131.2 | 0.003365925 | -0.352800129 | 0.174566175 | 0.702717632 | 0.499102624 | 0.989399868 | 0.043278644 |
| AP001160.3 | 0.011955243 | -0.214352624 | 0.084890548 | 0.807063745 | 0.683358928 | 0.953162184 | 0.011568281 |
| AL354696.1 | 0.045817331 | -0.640292746 | 0.249075002 | 0.527138084 | 0.323527623 | 0.85888975 | 0.010149834 |
| AL358472.3 | 0.036371701 | -0.297978543 | 0.111751952 | 0.742317268 | 0.596301812 | 0.924087291 | 0.007666201 |
| EGOT | 0.01068025 | -0.127044115 | 0.062929197 | 0.88069482 | 0.778502501 | 0.9963017 | 0.043503628 |
| AL136368.1 | 0.000572526 | -0.741931442 | 0.249068484 | 0.476193287 | 0.292264314 | 0.775873192 | 0.002893559 |
| AC008105.3 | 0.014447277 | -0.358813801 | 0.171744899 | 0.6985044 | 0.498861081 | 0.978044622 | 0.036687441 |
| AC098484.1 | 0.018128848 | 0.14692516 | 0.074283347 | 1.158267275 | 1.001333363 | 1.339796645 | 0.047939794 |
| TNFRSF14-AS1 | 0.000366053 | -0.602842862 | 0.204194509 | 0.547253656 | 0.366756644 | 0.816581155 | 0.003154193 |
| AC026401.3 | 0.048591097 | -0.041133496 | 0.019917858 | 0.959701005 | 0.922957787 | 0.997906982 | 0.038908185 |
| AC020907.4 | 0.003116858 | -0.339315263 | 0.152087773 | 0.712257865 | 0.528664206 | 0.959609637 | 0.025677888 |
| PRKCZ-AS1 | 0.034886091 | -0.436715624 | 0.150302918 | 0.646155156 | 0.481281024 | 0.8675108 | 0.00366585 |
| AC005840.4 | 0.019825969 | -0.367198536 | 0.168647859 | 0.692672111 | 0.497707728 | 0.964008848 | 0.029457505 |
| AC005104.1 | 0.040958468 | -0.351206827 | 0.161967152 | 0.703838166 | 0.512396463 | 0.966806369 | 0.030129555 |
| AC087741.1 | 0.006476471 | -0.209330462 | 0.098676176 | 0.811127145 | 0.668491089 | 0.98419748 | 0.033889158 |
| NIFK-AS1 | 0.000460273 | -0.457839073 | 0.150752342 | 0.632649278 | 0.470806436 | 0.850126674 | 0.002389233 |
| DLG5-AS1 | 0.010514569 | -0.180170874 | 0.076277075 | 0.835127498 | 0.719160159 | 0.969795015 | 0.018173799 |
| AC015819.1 | 0.011747394 | -0.283006818 | 0.129623833 | 0.75351465 | 0.584461211 | 0.971466227 | 0.029014233 |
| AL162274.2 | 0.035469257 | -0.299409691 | 0.128644113 | 0.741255662 | 0.57605766 | 0.953828052 | 0.019942588 |
