# Supplementary material for: A Novel Necroptosis-Associated lncRNA Signature Can Impact the Immune Status and Predict the Outcome of Breast Cancer
Source: J Immunol Res. 2022 May 5;2022:3143511. doi: 10.1155/2022/3143511 (PMC9107037; doi:10.1155/2022/3143511)
Supplement: Supplementary 3 — Table S3: multivariate Cox proportional hazard regression analysis results of necroptosis-related lncRNAs in BRCA. [file 3143511.f3.docx]

**Table S3 Mutivariate cox proportional hazard regression analysis results of necroptosis-related lncRNAs in BRCA.**

| gene | coef | HR | HR.95L | HR.95H | pvalue |
| --- | --- | --- | --- | --- | --- |
| AC010834.3 | 0.842324885 | 2.321758529 | 1.366690955 | 3.944244048 | 0.001837251 |
| AL031186.1 | -0.738263048 | 0.47794336 | 0.231700926 | 0.985882356 | 0.045667649 |
| AL136531.1 | -0.718556561 | 0.48745536 | 0.219837775 | 1.080854864 | 0.07696208 |
| LINC01871 | -0.442074244 | 0.642701917 | 0.453556797 | 0.910725529 | 0.012926285 |
| MAPT-AS1 | -0.492500228 | 0.611096602 | 0.405095804 | 0.921853676 | 0.018881172 |
| SEMA3B-AS1 | -0.306315799 | 0.736154106 | 0.556159837 | 0.974401298 | 0.032254966 |
| AL606834.2 | -0.453659669 | 0.635298908 | 0.340702972 | 1.18462337 | 0.153572982 |
