# Supplementary material for: A Novel Necroptosis-Associated lncRNA Signature Can Impact the Immune Status and Predict the Outcome of Breast Cancer
Source: J Immunol Res. 2022 May 5;2022:3143511. doi: 10.1155/2022/3143511 (PMC9107037; doi:10.1155/2022/3143511)
Supplement: Supplementary 4 — Table S4: clinical information variables for BRCA patients in the high- and low-risk groups. [file 3143511.f4.docx]

| Table S4 Clinical information variables for BRCA patients in the high-low risk group | | |
| --- | --- | --- |
| Variables | Low Risk Group(n=396) | High Risk Group(452) |
| Age（%） |  |  |
| <=60 | 233(58.8) | 261（57.7） |
| >60 | 163(41.2) | 191（42.3） |
| Stage（%） |  |  |
| 1 | 81(20.8) | 76(16.8) |
| 2 | 236(59.6) | 258(57.1) |
| 3 | 74(18.7) | 108(23.9) |
| 4 | 5(1.3) | 10(2.2) |
| T（%） |  |  |
| 1 | 117(29.5) | 114(25.2) |
| 2 | 226(57.1) | 268(59.3) |
| 3 | 46(11.6) | 49(10.8) |
| 4 | 7(1.8) | 21(4.6) |
| N（%） |  |  |
| 0 | 197(49.7) | 221(48.9) |
| 1 | 142(35.9) | 145(32.1) |
| 2 | 34(8.6) | 58(12.8) |
| 3 | 23(5.8) | 28(6.2) |
| M（%） |  |  |
| 0 | 391(98.7) | 442(97.8) |
| 1 | 5(1.3) | 10(2.2) |
| T, tumor; M, metastasis; N, lymph node. | |  |
