# Supplementary material for: A Novel Necroptosis-Associated lncRNA Signature Can Impact the Immune Status and Predict the Outcome of Breast Cancer
Source: J Immunol Res. 2022 May 5;2022:3143511. doi: 10.1155/2022/3143511 (PMC9107037; doi:10.1155/2022/3143511)
Supplement: Supplementary 5 — Table S5: Demographic and clinical characteristics of BRCA patients in TCGA, training set, and testing set. [file 3143511.f5.docx]

| Table S5 Demographic and clinical characteristics of BRCA patients in TCGA, training set and testing set | | | |
| --- | --- | --- | --- |
| Variables | Entire TCGA dataset (n = 848) | Training setn （n=427） | Testing setn （n=421） |
| Age（%） |  |  |  |
| <=60 | 494（58.3） | 238（55.7） | 256（60.8） |
| >60 | 354（41.7） | 189（44.3） | 165（39.2） |
| Stage（%） |  |  |  |
| 1 | 157（18.5） | 71（16.6） | 86（20.4） |
| 2 | 494（58.3） | 252（59） | 242（57.5） |
| 3 | 182（21.5） | 94（22） | 88（20.9） |
| 4 | 15（1.8） | 10（2.3） | 5（1.2） |
| T（%） |  |  |  |
| 1 | 231（27.2） | 110（25.8） | 121（28.7） |
| 2 | 494（58.3） | 262（61.4） | 232（55.1） |
| 3 | 95（11.2） | 42（9.8） | 53（12.6） |
| 4 | 28（3.3） | 13（3） | 15（3.6） |
| N（%） |  |  |  |
| 0 | 418（49.3） | 207（48.5） | 211（50.1） |
| 1 | 287（33.8） | 146（34.2） | 141（33.5） |
| 2 | 92（10.8） | 49（11.5） | 43（10.2） |
| 3 | 51（6） | 25（5.9） | 26（6.2） |
| M（%） |  |  |  |
| 0 | 833（98.2） | 417（97.7） | 396（98.8） |
| 1 | 15（1.8） | 10（2.3） | 5（1.2） |
| T, tumor; M, metastasis; N, lymph node. | | | |
