# Supplementary figures and images for: A Novel Necroptosis-Associated lncRNA Signature Can Impact the Immune Status and Predict the Outcome of Breast Cancer
Source: J Immunol Res. 2022 May 5;2022:3143511. doi: 10.1155/2022/3143511 (PMC9107037; doi:10.1155/2022/3143511)

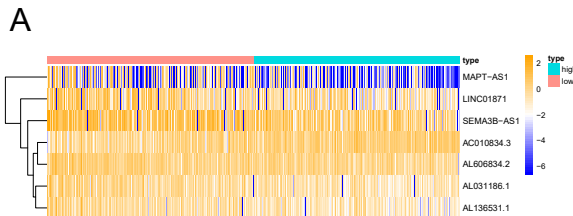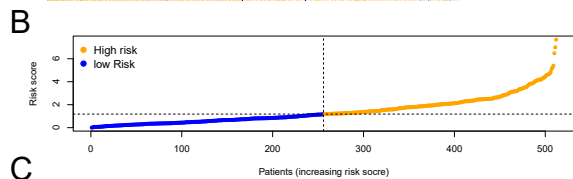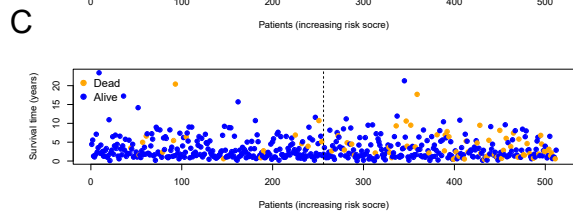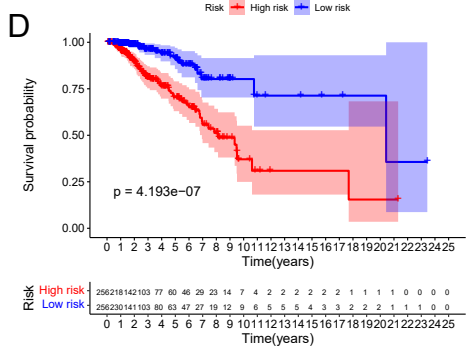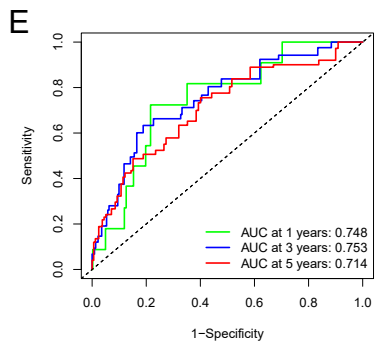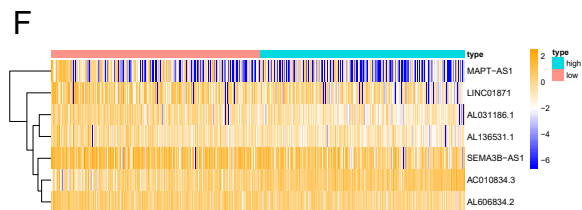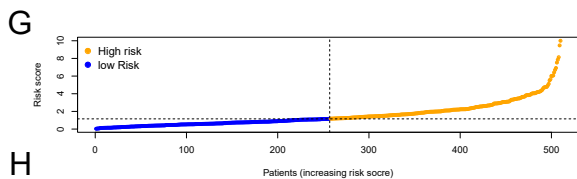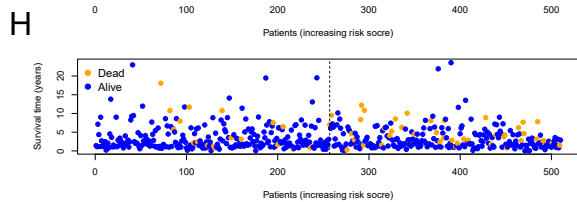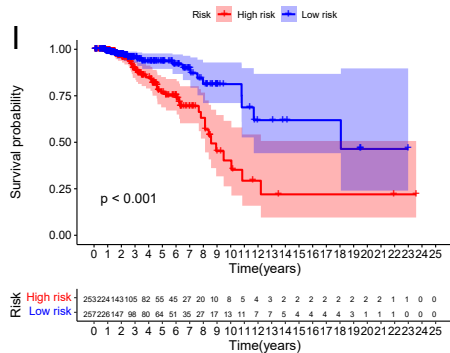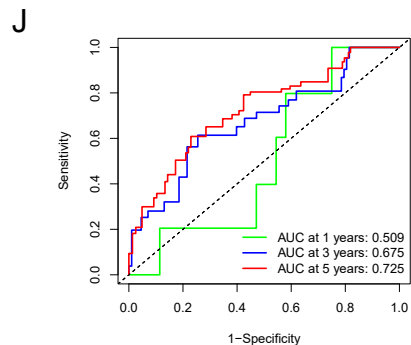

Supplement: Supplementary 6 — Supplement Figure 1: the training set and testing set are used to examine the veracity of the signature. (A) Kaplan-Meier analyzed OS rates in the high - and low-risk training set patients. (B) Heat map of expression of 7 lncRNAs in the training set. (C) Risk score distribution in training set patients. (D) Survival status of training set patients with different risk scores. (E) ROC curve and AUC value at 1-year, 3-year, and 5-year survival for the training set. (F) Kaplan-Meier analyzed OS rates in high- and low-risk testing set patients. (G) Heat map of expression of 7 lncRNAs in the testing set. (H) Risk score distribution in the testing set patients. (I) Survival status of the testing set patients with different risk scores. (J) ROC curve and AUC value at 1-year, 3-year, and 5-year survival for the testing set. [file 3143511.f6.pdf]
